# Being a Collective Jeremiah: The Academic Responsibility to Clarify How Not All Is Well

**DOI:** 10.1007/978-3-030-65355-2_6

**Published:** 2021-03-20

**Authors:** Erik Borgman

**Affiliations:** 1 Tilburg University, Tilburg, The Netherlands; 2 Tilburg University, Tilburg, The Netherlands; 3 Tilburg University, Tilburg, The Netherlands; 4 Tilburg University, Tilburg, The Netherlands; Tilburg School of Humanities and Digital Sciences, Tilburg, The Netherlands

## Abstract

COVID-19 has been frequently described as a great equalizer. The reality, however, is that long-standing inequities have been further exacerbated. The result is a lack of presence of a lot of stories on the impact of the coronavirus pandemic on societies and people. Thus speaks the website of Voice of Witness (https://voiceofwitness.org/unheard-voices-of-the-pandemic/, 2020), a San Francisco based organization with a mission to advance human rights “by amplifying the voices of people impacted by injustice.”

For obvious reasons, during the COVID-19 pandemic, public attention went almost exclusively to saving lives and overcoming problems in doing so. As a result, people felt their souls were left behind in the limbo of uncertainty without accompaniment.

In this short chapter, the starting point of Voice of Witness is taken: an understanding of any crucial issue is incomplete without deep listening and learning from people who have experienced it firsthand.

COVID-19 has been frequently described as a great equalizer. The reality, however, is that long-standing inequities have been further exacerbated. The result is a lack of presence of a lot of stories on the impact of the coronavirus pandemic on societies and people. Thus speaks the website of Voice of Witness (2020), a San Francisco based organization with a mission to advance human rights “by amplifying the voices of people impacted by injustice.” They are driven by a belief in “the transformative power of the story” and by the conviction “that an understanding of crucial issues is incomplete without deep listening and learning from people who have experienced injustice firsthand.”

## All Souls Matter

Recognizing that, to really understand the impact of COVID-19, we should focus on the untold and unheard stories about hidden lives means that we connect the pandemic to that other crisis that has come to the fore: systemic racism in Western culture. Following the killing of 44-year-old Georg Floyd by—now ex-police officer Derek Chauvin on May 25, 2020 in Minneapolis, we have seen a worldwide wave of protests, stating what should be obvious: black lives matter. The phrase not just implies that black lives should not be treated with the appalling contempt Chauvin showed by casually keeping his left hand in his trouser pocket while kneeling on Floyds neck and chocking him to death. The phrase also means black lives deserve to be fully noticed. As educationalist Parker Palmer (2016) wrote in a blog speaking from his own experience in a situation of clinical depression:

The human soul doesn’t want to be advised or fixed or saved. It simply wants to be witnessed—to be seen, heard and companioned exactly as it is. When we make that kind of deep bow to the soul of a suffering person, our respect reinforces the soul’s healing resources, the only resources that can help the sufferer make it through*.*

At Floyd’s memorial service, the congregation was silent for 8 min and 46 s, the time Chauvin had his knee on Floyd’s neck. Ritually, they made space for the untold story of his life and death. It is crucial to make this space part of the public sphere.

For obvious reasons, during the COVID-19 pandemic, public attention went almost exclusively to saving lives and overcoming problems in doing so. As a result, people felt their souls were left behind in the limbo of uncertainty without accompaniment. There was some marginal room in the media for people’s anxieties: losing their jobs or businesses, not being able to start college, not being allowed to visit the aging and the sick, or decently burying the death. But this is not the same as having the lived anxieties of the soul attended to (cf. Bennison 2020).

In this short chapter, I take the starting point of Voice of Witness: an understanding of any crucial issue is incomplete without deep listening and learning from people who have experienced it firsthand. We have hardly started to listen to our own stories as they resonate in our souls or even to realize that we have souls in which these stories resonate and that long to be seen, heard, and accompanied. Let alone that we have tried to hear the voices that are routinely neglected. This implies that our understanding of the COVID-19 pandemic is still hugely incomplete. First, I will explain that simply trying to move forward after the end of the lockdown is not a good idea. I will then argue that especially academic institutions have the responsibility to behave as collective Jeremiahs. We should take the risk of being mocked as “Terror is all around,” like Jeremiah was (Jer. 20: 10).

## The Centrality of Hope

With the lockdown lifted, there is a strong pressure to leave behind the experiences of anxiety and uncertainty. This is in line with a dominant tendency during the lockdown. Rich countries tried to generate trust by investing huge amounts of money in loans, guarantees, and even gifts to keep small, medium-sized, and big businesses from bankruptcy. The language was that of combat: we are at war and will be victorious. Leaders of poorer countries did not have the means to buy the trust of their citizens in this manner. Their strategy usually came down to simply bragging. In both cases is could be said, in Jeremiah’s words: “They have been treating the wound of my people carelessly, saying: ‘Peace, peace’, where there was no peace” (Jer. 6,13). For those in precarious situations, participating in the lockdown was not a realistic option. It is impossible for the homeless to stay at home! And, more broadly, for many, the choice between either depriving one’s family of even the basic necessities or taking the chance of being infected with SARS-CoV-2 was obvious. As a result, in some cases, they were fined or arrested for not submitting to regulations that were unable to protect them to begin with. Their only choice left was to survive by staying hopeful.

In affluent societies, there is a strong pressure to be optimistic in order to get the economy going again and to adapt smoothly to what in this book and elsewhere is called “the new normal.” This threatens the strategy of survival by staying hopeful, probably the only strategy not involving self-deception. Optimism is not the same thing as hope. In fact, it is something totally different. Vaclav Havel, then still a dissident Czech playwright, in an interview in the 1980s, clearly stated that hope is not “the conviction that something will turn out well, but the certainty that something makes sense, regardless of how it turns out.” This hope, Havel believes, gives us the strength to live and to try new things, even in conditions that seem as hopeless as they were in Central Europe under communist occupation.

Considering himself an agnostic, Havel (1990: 181–182) uses quasi-religious language in his description of hope:

It transcends the world that is immediately experienced, and is anchored somewhere beyond its horizons. I feel that its deepest roots are in the transcendental, just as the roots of human responsibility are, though of course I can’t—unlike Christians, for instance—say anything about the transcendental…

Hope frees the bearers of hope from the dictatorship of the possible and opens the gate for real change.

Following this line of thought, Czech psychologist, philosopher, and theologian Tomáš Halík, who worked with Havel and was converted to Roman Catholicism and clandestinely ordained a priest under communism, calls hope a crack in the supposed closeness of our reality “through which a ray of light from the future falls into the present.” Halík (2009) follows into the footsteps of French poet and essayist Charles Péguy (1873–1914), who considered hope an aspect of God in which the hope of people participates. Giving hope, God shirks human categories, breaks through human expectations, appeals to people in an unprecedented way, and makes them enter the realm of what, from the dominant perspectives, is impossible.

## The Prophetic Role of Universities

In order to gain hope, according to Halík, we first have to lose our false expectations—for instance, in our current case, that we can build a human society by simply adapting to “a new normal.” The latter will inevitably mean re-installing a society “that is hurting,” as Pope Francis (2016) said of our current one, a society “that is bleeding, and the price of its wounds normally ends up being paid by the most vulnerable.” Hope lives in the most vulnerable and their wounds require a healing that no restoration or adaption can provide. The pressure to be optimistic implies the pressure to silence their voices because, otherwise, they would be disturbing the illusionary peace. This is, however, exactly why these voices are important. The stories of the most vulnerable express what is silenced to enable the belief that our economies, societies, and cultures are free of systemic injustices. Thus, they represent hope that these injustices will be properly addressed and our commons, new and old, will truly become our common property.

Universities, therefore, will have to resist the call to optimism in order to foster true hope. It is impossible to elaborate here on what this would entail. But let us take some advice from Ignacio Ellacuría (1930–1989), who was the rector of the Universidad Centroamericana “José Simeón Cañas” (UCA) in El Salvador until he was assassinated together with five fellow Jesuits, their housekeeper, and her daughter. Following his mentor, Spanish philosopher Xavier Zubiri (1898–1983), Ellacuría considered humans sentient beings participating in reality. They have the responsibility to truly know their reality in order to judge in what state it is and to further the changes that announce themselves in it. This requires a university that is historical both in the sense of participating in history and of making history, in the view of Ellacuría (1975, 1982, 1989; cf. Lassalle-Klein 2014: 53–184; Hassett and Lacey (1991)). Pope Francis recently (Francis 2017: no. 4d; cf. Francis 2013: en no. 71) envisioned a university cultivating “a way of making history in a life setting where conflicts, tensions and oppositions can achieve the diversified and life-giving unity” needed to go forward.

We need the voices of protest and lament and have to be collective Jeremiahs in amplifying them. To fully know reality in its current historicity, special attention should be given to voices that are silenced and views that are disregarded; Ellacuría calls this “a preferential option for the poor.” Through their place in reality, the marginalized embody the hope for a change firmly rooted in the real and reaching out towards what seems impossible and what is unimaginable as yet. Thus, they open up a possible future in which black lives self-evidently matter, threats are addressed regardless of whom is threatened, and ways are searched and found to live with the SARS-CoV-2 virus and the new variants that will undoubtedly evolve, instead of constantly waging war against them. Ultimately, to be a Jeremiah in response to being chosen.
